# Supplementary material for: FastqPuri: high-performance preprocessing of RNA-seq data
Source: BMC Bioinformatics. 2019 May 3;20:226. doi: 10.1186/s12859-019-2799-0 (PMC6500068; doi:10.1186/s12859-019-2799-0)
Supplement: Supplementary file 2 — Archive of FastqPuri. Archive containing all files needed to install and run FastqPuri v1.0.6. Date stamp March 22, 2019. (GZ 47,819 kb) [file 12859_2019_2799_MOESM2_ESM.gz › FastqPuri-1.0.6/latex/struct__trimFilter_8h__dep__incl.pdf]

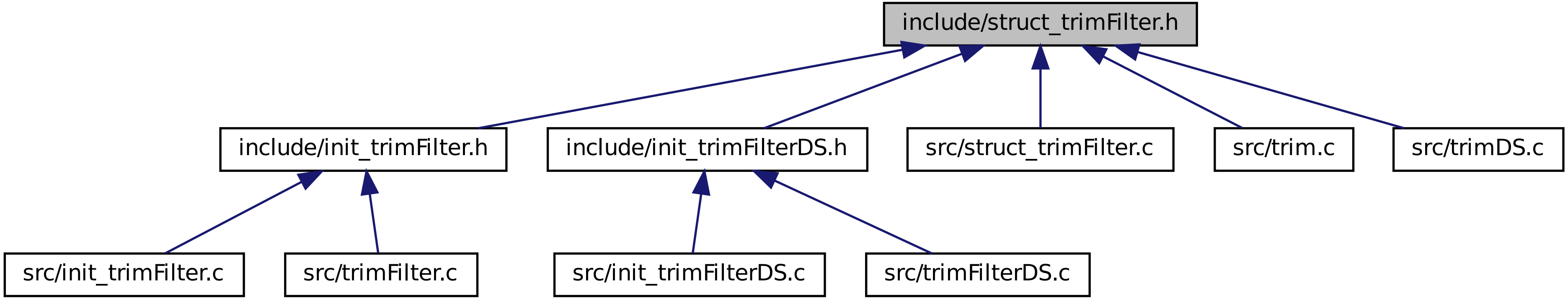
include/struct_trimFilter.h
include/init_trimFilter.h
include/init_trimFilterDS.h
src/struct_trimFilter.c
src/trim.c
src/trimDS.c
src/init_trimFilter.c
src/trimFilter.c
src/init_trimFilterDS.c
src/trimFilterDS.c
